# AlignmentViewer: Sequence Analysis of Large Protein Families

**DOI:** 10.12688/f1000research.22242.2

**Published:** 2020-10-15

**Authors:** Roc Reguant, Yevgeniy Antipin, Rob Sheridan, Christian Dallago, Drew Diamantoukos, Augustin Luna, Chris Sander, Nicholas Paul Gauthier

**Affiliations:** 1 Department of Informatics, Technical University of Munich, Munich, Germany; 2 cBio Center, Department of Data Sciences, Dana-Farber Cancer Institute, Boston, MA, USA; 3 Department of Cell Biology, Harvard Medical School, Boston, MA, USA; 4 Icahn School of Medicine, Mount Sinai, New York, NY, USA; 5 Knowledge Systems Group, Computational Oncology, Memorial Sloan Kettering Cancer Center, New York, NY, USA; 6 Broad Institute of MIT and Harvard, Cambridge, MA, USA

**Keywords:** alignment viewer, MSA, JavaScript, protein alignments, web-based, tool

## Abstract

AlignmentViewer is a web-based tool to view and analyze multiple sequence alignments of protein families. The particular strengths of AlignmentViewer include flexible visualization at different scales as well as analysis of conservation patterns and of the distribution of proteins in sequence space. The tool is directly accessible in web browsers without the need for software installation. It can handle protein families with tens of thousands of sequences and is particularly suitable for evolutionary coupling analysis, e.g. via EVcouplings.org.

## Introduction

Multiple sequence alignment (MSA) analysis (e.g., analysis of sequence patterns, subfamilies, specificity residues, evolutionary couplings) and visualization allows researchers to extract information and gain a better understanding of protein families. MSA is a basic step in many protein analysis workflows, including 3D structure prediction ( Marks *et al.*, 2011), structure detection in flexible (‘disordered’) domains ( Toth-Petroczy *et al.*, 2016), function prediction ( Tamames *et al.*, 1998) and intracellular localization ( Goldberg *et al.*, 2014).

A number of useful tools exist for the visualization of protein MSAs, such as, MView, Wasabi, AliView, MSAViewer and Jalview ( Brown *et al.*, 1998; Larsson, 2014; Veidenberg *et al.*, 2016; Waterhouse *et al.*, 2009; Yachdav *et al.*, 2016). MView was one of the first online browser-based MSA viewers, with alignments formatted as an HTML document. Wasabi is a web-based tool particularly useful for phylogenetic analysis and incorporates phylogeny-aware alignment methods. Another desktop application, AliView, has features such as sorting, viewing, removing, editing and merging sequences from large nucleotide sequence datasets. MSAViewer is an interactive MSA visualizer in JavaScript that implements basic features of viewing, scrolling and motif selection. Jalview is a Java-based desktop tool accessible through websites using an embeddable applet, but unfortunately the technology for these applets is no longer supported in most browsers.

AlignmentViewer complements these MSA tools and provides the following features: (i) in-browser and serverless execution, (ii) visualization of very large MSAs, (iii) visualization of conservation patterns, (iv) sequence filtering, (v) logo display, (vi) pairwise sequence identity map, (vii) sequence space exploration by UMAP dimensionality reduction, and (viii) display of top-ranked evolutionary couplings ( Hopf *et al.*, 2019).

An earlier version of this article can be found on bioRxiv (DOI: https://doi.org/10.1101/269720); additional features have been implemented since the earlier version.

## Methods

### Operation

AlignmentViewer is a web-based tool written in JavaScript with minimal system requirements. AlignmentViewer works best on Chrome regardless of operating system. AlignmentViewer is developed with the D3 library (d3js.org) to produce dynamic and interactive data visualizations, with performance (speed) for large alignments a major consideration. The tool is entirely client-based, running inside a web browser without the need for server-side computation.

### Implementation

Users can access AlignmentViewer and all its features directly from alignmentviewer.org, but its serverless execution enables anyone to quickly start a local copy for online or offline use. Hyperlinks for lookup in background databases, such as Uniprot or Pfam, are made directly from the client. Alignments can be passed to AlignmentViewer also via a URL query parameter that is served by https and is properly encoded (e.g., https://alignmentviewer.org/?url=https://alignmentviewer.org/example/1bkr_A.1-108.msa.txt), enabling seamless integration from external web services via a simple link (e.g. the EVcouplings, evcouplings.org, web server ( Hopf *et al.*, 2019) offers visualization of computed alignments via a link to AlignmentViewer). The tool has been thoroughly tested with many large alignments. An alignment with, e.g., 50,000 sequences (about 13 MB of memory) loads in the Safari browser within one minute; further speedup is planned.

## Use case

**Figure 1.**
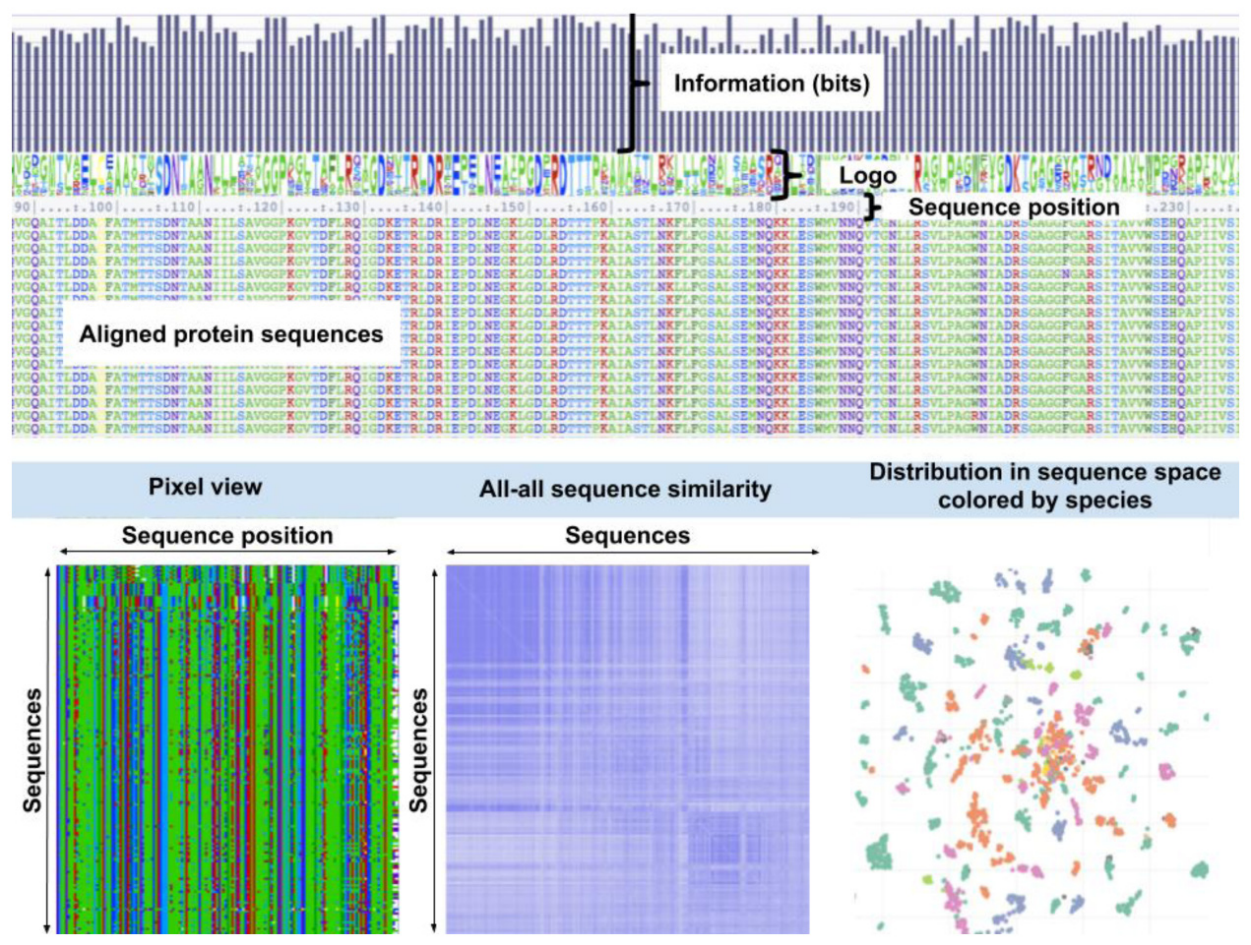
AlignmentViewer visualization of the beta-lactamase protein domain family. Bars above the sequence alignment quantify residue conservation. The alignment consensus logo (just below the bar chart) is based on the amino acid frequencies. Lower left: *pixel view* of the alignment especially useful for large families; lower middle: protein-protein sequence similarity matrix graded by percentage identity; lower right: distribution of sequences in sequence space (UMAP projection), colored by species groups.

Figure 1 shows the main functionalities from AlignmentViewer explained in more detail in the next subsections. The top sub-figure shows the *msa view* with the sequence logo and the alignment capturing most of the attention. This view lets the user examine in depth the alignment. Each amino acid position is represented in sequence logo and the height shows users the information content of each position, in bits. Then, from left to right we show the *pixel view*, a part of the *stats view*, and the sequence space (with annotations). The *pixel view* gives an overview display of the alignment to enable a coarse view of the alignment for better visualization and pattern identification. The all versus all sequence identity sub-figure in Figure 1 (part of the *stats view*) displays allows users to identify possible clusters in the alignment based on sequence identity. The bottom right sub-figure of Figure 1 displays the sequences clustered by similarity (see section *Sequence space*) highlighted by user-provided annotations to aid in interpretation of the clusters.

### MSA view

***Alignment details.*** The *msa view* page has summary information: number of sequences, conservation and gap counts for each position, a sequence logo, and the residues in one letter code. By default, columns with gaps in the reference sequence (first row) are omitted in order to facilitate visual focus on sequence patterns relative to a protein of interest and to avoid extremely gapped alignment views typical of many MSA presentations. The amino acids are colored using a conventional coloring scheme, adopted from Mview, based on amino acid properties.

***Sequence attributes and sorting.*** Sequences in the alignment can be sorted using one of four different methods: (i) the original order provided by the user, (ii–iii) by % sequence identity between a particular sequence and the reference (top) sequence, relative to the first or the second (gaps not counted), and (iv) by user-provided (upload annotations tab) sequence weights or other attributes, such as alignment profile scores (e.g., HMM bit scores). Sequences can be filtered by sequence identity relative to a reference sequence or by percentage of gaps.

### Pixel view (suitable for large families)

The *pixel view* ( *image view* website tab) leads to an overview of the entire depth and breadth of an MSA. The amino acid letters are represented by small rectangles of pixels, retaining the amino acid type coloring ( *image view* tab). This striking visual impression can reveal patterns of conservation and variation, especially for large alignments. This is very useful to gain an intuitive view of sequence properties, noise at the uncertain edges of a protein family, as well as subfamily distributions. The coloring scheme can be by (1) amino acid properties, (2) hydrophobicity (red to blue) or (3) mutational difference (stronger color) in a sequence relative to the reference (first row) sequence.

### Stats view

The *stats view* tab leads to plots of statistical properties of the set of sequences in the alignment, including (i) sequence identity relative to the reference sequence, and (ii) min, max, and average of (i); and (iii) a pairwise sequence identity matrix in which each pixel represents the degree of similarity between two sequences, such that a block-diagonal structure of the matrix is indicative of distinct subfamilies, given, e.g., a tree-derived sequence order as user input. The ordering of sequences by phylogeny is (currently) not part of the tool and can be performed using external tools, e.g., Wasabi ( Veidenberg *et al.*, 2016).

### Annotations and evolutionary couplings

Users can upload custom numerical attributes or labels for the sequences in the MSA ( *upload annotations*) or evolutionary couplings between residue positions ( *load couplings*). Adding these attributes allows users to use sequence weights, compare different measures of sequence fitness (e.g., bitscore, sequence identity, statistical energy) or visualize evolutionary coupling constraints for pairs of positions.

### Sequence space

Users can view representations of the MSA sequences in two- or three-dimensional space under the “sequence space” tab. These representations are generated using the Uniform Manifold Approximation and Projection (UMAP) dimensionality reduction algorithm ( McInnes *et al.*, 2018), whichhas been adapted for Javascript using the umap-js library ( https://github.com/PAIR-code/umap-js). The alignmentviewer.org implementation uses the number of amino acid differences between pairs of sequences (the Hamming distance) as the distance metric parameter. The algorithm then iteratively calculates an embedding in two- or three-dimensional space, which is displayed in real time for the end users. UMAP hyperparameters are set to reasonable defaults, but can also be configured via the settings panel. Sequences can be colored by user provided annotations ("upload annotations" tab).

## Conclusion

AlignmentViewer is a lightweight online viewer for biological multiple sequence alignments that focuses on usability and performance. Written in JavaScript, this tool can be used in many browsers. The architecture of AlignmentViewer allows its use without software installation and without an internet connection. The visualization capabilities, analysis features and metrics in AlignmentViewer are useful in many areas of biology, especially evolutionary, structural, synthetic and chemical biology. In the future we plan to add a visualization of species diversity, predicted contact maps, and organization by sequence subfamilies with specificity residues. A standalone version of AlignmentViewer is available at alignmentviewer.org and is in use by external services including EVcouplings.org. AlignmentViewer is an open source project hosted on GitHub, which welcomes engagement of interested members of the community.

## Data availability

All data underlying the results are available as part of the article and no additional source data are required.

## Software availability

**AlignmentViewer website and demo can be found at:** https://alignmentviewer.org/.

**Source code available at:** https://github.com/sanderlab/alignmentviewer.

**Archived source code at time of publication:** https://doi.org/10.5281/zenodo.4063551 ( Reguant, 2020).

**License:** MIT license.
