# Supplementary material for: AlignmentViewer: Sequence Analysis of Large Protein Families
Source: F1000Res. 2020 Oct 15;9:ISCB Comm J-213. Originally published 2020 Mar 27. [Version 2] doi: 10.12688/f1000research.22242.2 (PMC7570326; doi:10.12688/f1000research.22242.2)
Supplement: Peer review history

# Peer review: Reviewer response for version 2

**DOI:** 10.5256/f1000research.30135.r73164

**Published:** 2020-10-16

**Authors:** Alex Bateman

**Affiliations:** 1 European Molecular Biology Laboratory, European Bioinformatics Institute (EMBL-EBI), Wellcome Genome Campus, Hinxton, UK

The authors have addressed my concerns fully.

 The authors may wish to double check the alignment of the example 1KR:A on the website. It does not look well aligned to my eyes. This suggestion is not required for approval of the manuscript.

Are the conclusions about the tool and its performance adequately supported by the findings presented in the article?

Partly

Is the rationale for developing the new software tool clearly explained?

Yes

Is the description of the software tool technically sound?

Yes

Are sufficient details of the code, methods and analysis (if applicable) provided to allow replication of the software development and its use by others?

Partly

Is sufficient information provided to allow interpretation of the expected output datasets and any results generated using the tool?

Yes

Reviewer Expertise:

Computational biology with specialism in biological databases and analysis of protein families.

I confirm that I have read this submission and believe that I have an appropriate level of expertise to confirm that it is of an acceptable scientific standard.

# Peer review: Reviewer response for version 1

**DOI:** 10.5256/f1000research.24533.r61763

**Published:** 2020-04-16

**Authors:** Alex Bateman

**Affiliations:** 1 European Molecular Biology Laboratory, European Bioinformatics Institute (EMBL-EBI), Wellcome Genome Campus, Hinxton, UK

Overall there is a need for Javascript based alignment viewers which can handle large numbers of sequences. So this software is rather timely. However, I think the current implementation seems to still contain significant bugs and the web page requires a round of user experience testing to make it easier to use and interpret the results. Once these improvements are made then I think this has the potential to be a very useful tool.

**Manuscript changes:**

 Please fix capitalisation of PFAM to Pfam

**Software/Website changes:**

 The computing conservation box is annoyingly placed. It does not go away and covers the UMAP view significantly.

 I really need an estimate of how long it will take for the pairwise identity to load. Some time passes…I didn’t realise I had to click on calculate to get the pairwise identity to show. Please UX test this page to make it easier/clearer for the user to understand what to do.  

 For the top graph on the stats view the three colours used for max/average and min identity are hard to distinguish. Please use a broader colour palette. Please add axis legends for this graph and make it clearer what the title of this graph is. The title is very clear for the bottom plot.

 Please add alternative colouring schemes for viewing the alignments. It makes a surprisingly big difference for experienced users ability to interpret an alignment. I personally like the ClustalX colouring scheme that is widely adopted, but there are other popular schemes it would be nice to incorporate as options.

 “Based on this and other small tests we can safely state that any computer that is able to run any modern web browser will be able to run any alignment that requires visualization”. This is overstating the case. Needs to be toned down. Try testing the software on the ABC transporter family (PF00005) alignment in Pfam. The NCBI alignment contains 2.6 million sequences. If it works seamlessly for that then you can safely state.

 I tried to hook the viewer up to a Pfam alignment which is not very large (840 sequences). I got a box that said Fetching file…and no response beyond that.

 https://alignmentviewer.org/?url=http://pfam.xfam.org/family/PF00571/alignment/seed/format?format=fasta&alnType=seed&order=t&case=l&gaps=default&download=0

 also tried Stockholm format with no luck.

 https://alignmentviewer.org/?url=http://pfam.xfam.org/family/PF00571/alignment/seed/format?format=stockholm&alnType=seed&order=t&case=l&gaps=default&download=0

 I also tried with a Pfam family with just 3 sequences. Still with no luck.

 I got the viewer to work by uploading a Pfam alignment in fasta format with 3 sequences. Then I tried uploading the seed alignment for the CBS domain downloaded from Pfam in fasta format. I then got the following error:

 Parsing error: sequence #3 has different length.

 The first few lines of the alignment are included below and the third sequence appears to be the same length as all the others.

 >Q8EHI4_SHEON/79-134

 I........H.Q...V........MTR........NP.........V.......TVAPY.

 ...VS.LDVAS....RT..L.....L....E.....HN..I.....G..CL.P.V.L...

 .........ENG....D......LV.GIVTWKDLLRAYCA

 >Q97V95_SULSO/193-248

 V........L.D...A.......G.TK........NP.........I.......TINRY.

 ...YS.ILNAA....KL..M.....I....E.....KR..I.....G..TL.L.V.M...

 .......E..NQ....K......LV.GIVTERDLMYAYIN

 >Q6M020_METMP/269-321

 V........K.E...I.........MS........PP.........V.......MVSPE.

 ...AA.LNELI....KG..M.....A....N.........T.....D..RV.Y.V.V...

 .........DNG....N......IL.GIISKTDIVRTLSI

 >Q72KI1_THET2/367-424

 V........E.G...F.........LA........RA.........V.......VLPPS.

 ...TP.LSQVE....PR..L..........R.....EA..G.....G..RV.L.V.GE..

 .......RAGEG...WR......LL.GIYTRTDLYRSAPK

 >Q8TX96_METKA/244-298

 A........R.N...Y.......L..Q........EM.........V.......VVPPE.

 ...TP.LHEAL....WE..V.........ID.....KM..S.....D..RI.Y.V.M...

 ..........DG.....R....KLT.GVVPLIDAVYTLAK

 >Q836T2_ENTFA/77-132

 V........Q.E...I........MSP........PL.................MVAQD.

 ...TS.IRDAI....TN..L................FMYDV.....G..SL.Y.V.M...

 ........DEAK....E......LL.GVLSRKDLLRASLN

 >Q8XIV1_CLOPE/77-131

 V........K.D...I.........MS........KP.........V.......TVCEE.

 ...TM.LHDAI....VH.LF.....L...ND.........V.....G..TM.F.VEN...

 ..........GG....V......LT.GAVSRKDFLKVAIG

 If I delete the third sequence and re-upload I get exactly the same error.

 For info I carried out tests with Firefox for Mac version 74.0 and Safari Version 13.1

Are the conclusions about the tool and its performance adequately supported by the findings presented in the article?

Partly

Is the rationale for developing the new software tool clearly explained?

Yes

Is the description of the software tool technically sound?

Yes

Are sufficient details of the code, methods and analysis (if applicable) provided to allow replication of the software development and its use by others?

Partly

Is sufficient information provided to allow interpretation of the expected output datasets and any results generated using the tool?

Yes

Reviewer Expertise:

Computational biology with specialism in biological databases and analysis of protein families.

I confirm that I have read this submission and believe that I have an appropriate level of expertise to confirm that it is of an acceptable scientific standard, however I have significant reservations, as outlined above.

# Response

**Published:** 2020-10-09

**Authors:** Roc Reguant

**Affiliations:** Dana-Farber Cancer Institute and Harvard Medical School, USA

*Manuscript changes:*

*Please fix capitalisation of PFAM to Pfam*

**Response: **The manuscript has been updated to Pfam.

*Software/Website changes:*

*The computing conservation box is annoyingly placed. It does not go away and covers the UMAP view significantly.*

**Response: **This bug has now been fixed - the pop-up will disappear after the alignment has been loaded.

*I really need an estimate of how long it will take for the pairwise identity to load. Some time passes…I didn’t realise I had to click on calculate to get the pairwise identity to show. Please UX test this page to make it easier/clearer for the user to understand what to do.*

**Response: **We have added a button into the middle of the plot to indicate that an action needs to be taken by the user to begin calculating pairwise sequence identity.

*For the top graph on the stats view the three colours used for max/average and min identity are hard to distinguish. Please use a broader colour palette. Please add axis legends for this graph and make it clearer what the title of this graph is. The title is very clear for the bottom plot.*

**Response: **We have updated the color palette to increase the contrast, added axis labels and added a clear title. 

*Please add alternative colouring schemes for viewing the alignments. It makes a surprisingly big difference for experienced users ability to interpret an alignment. I personally like the ClustalX colouring scheme that is widely adopted, but there are other popular schemes it would be nice to incorporate as options.*

**Response: **We agree alternative coloring schemes are useful and have added the clustal colors as a drop-down option on the main page as well as in the image view.

*“Based on this and other small tests we can safely state that any computer that is able to run any modern web browser will be able to run any alignment that requires visualization”. This is overstating the case. Needs to be toned down. Try testing the software on the ABC transporter family (PF00005) alignment in Pfam. The NCBI alignment contains 2.6 million sequences. If it works seamlessly for that then you can safely state.*

**Response: **We agree with the reviewer that the phrasing was optimistic and have removed the sentence from the main manuscript.

*I tried to hook the viewer up to a Pfam alignment which is not very large (840 sequences). I got a box that said Fetching file…and no response beyond that.*

*https://alignmentviewer.org/?url=http://pfam.xfam.org/family/PF00571/alignment/seed/format?format=fasta&alnType=seed&order=t&case=l&gaps=default&download=0*

*also tried Stockholm format with no luck.*

*https://alignmentviewer.org/?url=http://pfam.xfam.org/family/PF00571/alignment/seed/format?format=stockholm&alnType=seed&order=t&case=l&gaps=default&download=0*

**Response: **There are two technical limitations inherent to how requests happen in the browser. We take as an example your suggested URL: https://alignmentviewer.org/?url=http://pfam.xfam.org/family/PF00571/alignment/seed/format?format=stockholm&alnType=seed&order=t&case=l&gaps=default&download=0

Limitation 1: The alignmentviewer.org website is hosted as a secure site under https. However, the location of the requested alignment (from Pfam) is passed via an insecure connection (http). For security reasons mixing different protocols (also called "mixed content", see https://developer.mozilla.org/en-US/docs/Web/Security/Mixed_content) is not allowed.

Limitation 2: the string passed to the query parameter URL (aka. what follows "?url=") needs to be sanitized before being served. More information about this can be found here: https://en.wikipedia.org/wiki/Query_string#URL_encoding. For clarity, we have updated the example in the main text to be stanitized / encoded.  

In your example, the alignment lives at " http://pfam.xfam.org/family/PF00571/alignment/seed/format?format=stockholm&alnType=seed&order=t&case=l&gaps=default&download=0". Passing the string as-is will confuse the browser that will interpret e.g. "&gaps=" as query for alignmentviewer rather than for pfam.xfam.org . Many languages offer built-in functions to sanitize strings to be passed to URL queries (e.g. in a JavaScript frontend application, using the window.encodeURIComponent() function; an interactive website can be found here: https://www.url-encode-decode.com). By doing so, the string proposed for the alignment would result in: "http%3A%2F% 2Fpfam.xfam.org%2Ffamily%2FPF00571%2Falignment%2Fseed%2Fformat%3Fformat%3Dstockholm%26alnType%3Dseed%26order%3Dt%26case%3Dl%26gaps%3Ddefault%26download%3D0".

Conclusion: considering the two inherit browser limitations mentioned above, your requests can be sanitized and fixed as follows:

https://alignmentviewer.org/?url=https%3A%2F%2Fpfam.xfam.org%2Ffamily%2FPF00571%2Falignment%2Fseed%2Fformat%3Fformat%3Dfasta%26alnType%3Dseed%26order%3Dt%26case%3Dl%26gaps%3Ddefault%26download%3D0

Again, note that for both URLs the protocol has been changed to "https" instead of "http", and that the string that follows "&url=" has been sanitized using Chrome's built-in console and the "window.encodeURIComponent(x)" function, where x is the string to be sanitized. We have added some text to the manuscript to highlight these requirements.

*I also tried with a Pfam family with just 3 sequences. Still with no luck.*

*I got the viewer to work by uploading a Pfam alignment in fasta format with 3 sequences. Then I tried uploading the seed alignment for the CBS domain downloaded from Pfam in fasta format. I then got the following error:*

*Parsing error: sequence #3 has different length.*

*The first few lines of the alignment are included below and the third sequence appears to be the same length as all the others.*

*>Q8EHI4_SHEON/79-134*

*I........H.Q...V........MTR........NP.........V.......TVAPY.*

*...VS.LDVAS....RT..L.....L....E.....HN..I.....G..CL.P.V.L...*

*.........ENG....D......LV.GIVTWKDLLRAYCA*

*>Q97V95_SULSO/193-248*

*V........L.D...A.......G.TK........NP.........I.......TINRY.*

*...YS.ILNAA....KL..M.....I....E.....KR..I.....G..TL.L.V.M...*

*.......E..NQ....K......LV.GIVTERDLMYAYIN*

*>Q6M020_METMP/269-321*

*V........K.E...I.........MS........PP.........V.......MVSPE.*

*...AA.LNELI....KG..M.....A....N.........T.....D..RV.Y.V.V...*

*.........DNG....N......IL.GIISKTDIVRTLSI*

*>Q72KI1_THET2/367-424*

*V........E.G...F.........LA........RA.........V.......VLPPS.*

*...TP.LSQVE....PR..L..........R.....EA..G.....G..RV.L.V.GE..*

*.......RAGEG...WR......LL.GIYTRTDLYRSAPK*

*>Q8TX96_METKA/244-298*

*A........R.N...Y.......L..Q........EM.........V.......VVPPE.*

*...TP.LHEAL....WE..V.........ID.....KM..S.....D..RI.Y.V.M...*

*..........DG.....R....KLT.GVVPLIDAVYTLAK*

*>Q836T2_ENTFA/77-132*

*V........Q.E...I........MSP........PL.................MVAQD.*

*...TS.IRDAI....TN..L................FMYDV.....G..SL.Y.V.M...*

*........DEAK....E......LL.GVLSRKDLLRASLN*

*>Q8XIV1_CLOPE/77-131*

*V........K.D...I.........MS........KP.........V.......TVCEE.*

*...TM.LHDAI....VH.LF.....L...ND.........V.....G..TM.F.VEN...*

*..........GG....V......LT.GAVSRKDFLKVAIG*

*If I delete the third sequence and re-upload I get exactly the same error.*

*For info I carried out tests with Firefox for Mac version 74.0 and Safari Version 13.1*

**Response: **Thank you for pointing out this issue. This bug occurred when refreshing an internal variable, and has now been fixed.

# Peer review: Reviewer response for version 1

**DOI:** 10.5256/f1000research.24533.r61767

**Published:** 2020-04-09

**Authors:** Erik Larsson Lekholm

**Affiliations:** 1 Department of Medical Biochemistry and Cell Biology, Institute of Biomedicine, The Sahlgrenska Academy, University of Gothenburg, Gothenburg, Sweden

This Software Tool article, describing a web-based sequence alignment viewer (AlignmentViewer), is well-written and very clearly presented. The introduction nicely motivates the need for this tool, given the many other available alternatives. The main features of the software are clearly explained in the article. The tool in itself seems useful and ran quickly and efficiently when tested on sample data. I have only a few minor comments that may be considered by the authors:

**Minor:**

- A little bit of detail may be added to the “Sequence space” section. “Two- or three-dimensional sequence space” is at first a bit confusing, as no information has been given at that stage as to what it means. How is UMAP applied to the Hamming distances? Is each sequence first represented by a vector of Hamming distances, with each element corresponding to one of the included sequences? Maybe this can be clarified with a few additional words.
- The following sentence is somewhat confusing: " Figure 1 shows the main functionalities from AlignmentViewer ( Reguant, 2020) explained in more detail in the next subsections.”
- "This view lets the user examine in depth the alignment.” - should it be “examine the alignment in depth"?
- When loading an alignment using a somewhat older version of Safari (12.1.2), the “computing conservation” window does not disappear after the operation finishes. This problem was absent in Chrome.

Are the conclusions about the tool and its performance adequately supported by the findings presented in the article?

Yes

Is the rationale for developing the new software tool clearly explained?

Yes

Is the description of the software tool technically sound?

Yes

Are sufficient details of the code, methods and analysis (if applicable) provided to allow replication of the software development and its use by others?

Yes

Is sufficient information provided to allow interpretation of the expected output datasets and any results generated using the tool?

Yes

Reviewer Expertise:

Genomics, bioinformatics

I confirm that I have read this submission and believe that I have an appropriate level of expertise to confirm that it is of an acceptable scientific standard.

# Response

**Published:** 2020-04-29

**Authors:** Chris Sander

**Affiliations:** Dana-Farber Cancer Institute and Harvard Medical School, USA

Thank you for your comments and suggestions - they are spot on.

We will work on taking these into account on the web site and in the next version of the manuscript.

Chris Sander - DFCI and HMS

# Response

**Published:** 2020-10-09

**Authors:** Roc Reguant

**Affiliations:** Dana-Farber Cancer Institute and Harvard Medical School, USA

*A little bit of detail may be added to the “Sequence space” section. “Two- or three-dimensional sequence space” is at first a bit confusing, as no information has been given at that stage as to what it means. How is UMAP applied to the Hamming distances? Is each sequence first represented by a vector of Hamming distances, with each element corresponding to one of the included sequences? Maybe this can be clarified with a few additional words.*

**Response: We agree that a bit more detail is warranted and have updated the manuscript that describes using the hamming distance between individual sequences as the distance metric (parameter) for UMAP.**

*The following sentence is somewhat confusing: "Figure 1 shows the main functionalities from AlignmentViewer (Reguant, 2020) explained in more detail in the next subsections.”*

**Response: We have updated the manuscript to make it more understandable. The citation to Reguant 2020 was also confusing (it points to the codebase) so we moved that codebase DOI to a single instance at the end of the manuscript.**

****

*"This view lets the user examine in depth the alignment.” - should it be “examine the alignment in depth"?*

**Response: The manuscript has been corrected.**

****

*When loading an alignment using a somewhat older version of Safari (12.1.2), the “computing conservation” window does not disappear after the operation finishes. This problem was absent in Chrome.*

**Response: Thank you for noticing this bug. We have now corrected the issue and the dialog disappears when using Safari.**
